# Factors associated with relapse and hospital death in patients coinfected with visceral leishmaniasis and HIV: a longitudinal study

**DOI:** 10.1186/s12879-023-08009-1

**Published:** 2023-03-07

**Authors:** Larissa D. L. N. Costa, Uiara S. Lima, Vandilson Rodrigues, Mayara I. S. Lima, Lucilene A. Silva, Jorim Ithamar, Conceição M. P. S. Azevedo

**Affiliations:** 1 Health Sciences Graduate Program, Federal University of Maranhão, Avenida dos Portugueses, 1966, Bacanga, São Luís, Maranhão 65080-806 Brazil; 2 Department of Morphology, Federal University of Maranhão, São Luís, Maranhão Brazil; 3 Health and Environment Graduate Program, Federal University of Maranhão, São Luís, Maranhão Brazil; 4 President Vargas State Hospital, São Luís, Maranhão Brazil; 5 Department of Medicine, Federal University of Maranhão, São Luís, Maranhão Brazil

**Keywords:** Visceral leishmaniasis, HIV, Recurrence, Death, Brazil

## Abstract

**Objective:**

Visceral leishmaniasis (VL) is an endemic parasitic disease in Latin America, and its clinical picture is aggravated in coinfections with the human immunodeficiency virus (HIV). The objective of this study was to investigate clinical factors and laboratory variables associated with VL relapse and death in VL/HIV coinfected patients.

**Methods:**

A prospective longitudinal study was conducted from January 2013 to July 2020 among 169 patients coinfected with VL and HIV. The outcomes investigated were the occurrence of VL relapse and death. Chi-square test, Mann–Whitney test and logistic regression models were used for statistical analysis.

**Results:**

The occurrence rates were 41.4% for VL relapse and 11.2% for death. Splenomegaly and adenomegaly were associated with the increased risk of VL relapse. Patients with VL relapse had higher levels of urea (*p* = .005) and creatinine (*p* < .001). Patients who died had lower red blood cell counts (*p* = .012), hemoglobin (*p* = .017) and platelets (*p* < .001). The adjusted model showed that antiretroviral therapy for more than 6 months was associated with a decrease in VL relapse, and adenomegaly was associated with an increase in VL relapse. In addition, edema, dehydration, poor general health status, and paleness were associated with an increase in hospital death.

**Conclusion:**

The findings suggest that adenomegaly, antiretroviral therapy, and renal abnormalities can be associated with VL relapse, while hematological abnormalities, and clinical manifestations like paleness, and edema can be associated with an increased odds of hospital death.

*Trial registration number:* The study was submitted to the Ethics and Research Committee of the Federal University of Maranhão (Protocol: 409.351).

## Introduction

Visceral leishmaniasis (VL) is an infectious disease caused by protozoa of the genus *Leishmania* [1]. It occurs mainly in tropical and subtropical areas, with higher rates in populations that are in situations of social vulnerability. In Latin America, 97% of VL cases occurred in Brazil in 2019. Between 2009 and 2019, 40,326 cases were reported in Brazil, 74.8% of them were distributed in seven states of the country (Minas Gerais, Maranhão, Ceará, Pará, Bahia, Tocantins and Piauí), and five of them are located in the northeastern region of this country [2, 3].

VL cases are becoming urbanized as people leave rural areas. Meanwhile, other diseases such as the Human Immunodeficiency Virus (HIV) are advancing, as they are leaving large cities and also becoming incident in rural areas. Therefore, reports of cases of VL/HIV coinfection are starting to emerge [4, 5]. Between the years of 2009 and 2019, 3,459 cases of leishmaniasis were reported in HIV-infected individuals in Brazil, which may mean a coinfection rate of about 6% [3, 6, 7]. Maranhão state, which is located in northeastern Brazil, ranked second in the country in the number of cases of VL/HIV coinfection from 2007 to 2017 and accounted for 11.9% and 11% of the country's cases, respectively [3, 4].

VL/HIV coinfection induces an increase in the lethality rate and an increase in the number of visceral leishmaniasis relapses, in proportions of approximately threefold and fivefold respectively, compared with HIV-negative groups. In addition, it favors the appearance of unusual clinical manifestations [5]. Some evidences have shown that coinfected patients develop a chronic immune activation, with reduced responses to therapy, presenting higher frequencies of clinical manifestation, higher chances of relapse, and mortality [4].

In this context, understanding the clinical features and serum markers associated with VL relapses and deaths in VL/HIV coinfection may help to identify patients more susceptible to adverse outcomes and guide therapy and monitoring. Therefore, the present study aimed to investigate predictive factors for VL relapse and hospital death among patients coinfected with VL/HIV.

## Methods

### Study design and sample selection

A prospective longitudinal study was conducted with the coinfected patients who were admitted from the year 2013 to 2019 in the hospital unit where the research was conducted. These patients were treated for the coinfection, being reassessed after 12 months for analysis of the outcome. Clinical evaluation was performed along with the search for laboratory information in the medical record. It should be noted that from the year 2020 to 2022, due to the pandemic situation, it was not possible to continue the study. The present study was conducted in a reference hospital for the treatment of infectious-parasitic diseases in the city of São Luís, state of Maranhão, Brazil. The present study was approved by the Research Ethics Committee of the University Hospital of Maranhão.

The study sample included 169 patients of both sexes, older than 18 years of age, who were serologically positive for HIV and had a parasitological diagnosis of VL, determined from bone marrow aspirates, according to Brazilian Ministry of Health guidance [8, 9]. These patients were treated for coinfection in the referral hospital. The exclusion criteria were the lack of follow-up treatment at the hospital and incompleteness of laboratory records [3].

### Data collection

Clinical and laboratory data were collected at the time of admission of the patient with a confirmed diagnosis of HIV and strong suspicion of VL. The outcomes investigated in this study were VL relapse and death during treatment follow-up. The definitions adopted for cure, relapse, and death followed the guidelines stipulated by the Brazilian Ministry of Health. Clinical recovery was considered when parasite suppression was maintained for a period longer than 12 months. Relapse was defined as the return of symptoms after clinical recovery. Death was defined as death after confirmation of the VL diagnosis of the patients [5]. All patients were followed up for at least 12 months after treatment for the coinfection.

Exposure and severity (KalaCal) variables [10] were collected based on the "visceral leishmaniasis death investigation form", which is an instrument used by the Brazilian Ministry of Health for VL surveillance and control actions [9]. Data included VL history (previous disease and treatment used), physical assessment (hydration status, abdominal protrusion, hepatosplenomegaly, and edema), HIV and leishmaniasis therapy, laboratory tests (complete blood count, AST, ALT, urea, creatinine, glycemia, CD4 T lymphocyte count, viral load and myelogram) and evolution (relapse, death or hospital discharge).

The presence of hepatomegaly was evaluated by palpating the liver and measuring its size using the Adams Classification. The occurrences of hepatomegaly were considered mild if the liver length exceeded 1 to 2 cm, were considered moderate if there was a 3 to 7 cm increase, and were considered as major increase if the liver length was greater than 7 cm increase [11].

The spleen was evaluated by palpation and percussion. The occurrences of splenomegaly were considered when the spleen was palpable or with the presence of flank bulging. Changes in the spleen were considered grade I (spleen palpable only below the costal margin), grade II (spleen palpable between the costal margin and a line across the umbilical scar), and grade III (spleen palpable below the umbilical scar). To qualify the state of pallor, the color of the mucous membranes was examined through quantitative evaluation using a scale from 1 to 4, such that + represented mild or discrete pallor; +  + and +  +  + , moderate pallor; and +  +  +  + severe pallor [11].

Clinical and laboratory scores were calculated by adding up the scores obtained for the presence of clinical and laboratory findings (Table 1). For this, age from 20 to 40 years was considered as 1 point and over 40 years 02 points in the severity score. The presence of bleeding was also considered with 1 point for bleeding in up to two different sites, 2 points for bleeding from three to four sites, and 3 points in cases where there was bleeding in more than five sites. The signs of edema, jaundice, dyspnea and bacterial infection were scored as 1 point for each sign present. Leukopenia was scored as 2 points and the presence of AIDS (Acquired Immune Deficiency Syndrome), thrombocytopenia, and renal insufficiency as 03 points each. Patients with a score greater than or equal to six, in clinical and laboratory criteria, are those who are at increased risk of progressing to death. These scores are used to monitor the health status of VL patients in Brazil [12].

**Table 1:** Prognostic models were built by adding clinical and laboratory variables that are used by the Brazilian Ministry of Health for monitoring patients with visceral leishmaniasis

Presence of factors	Clinical score	Laboratory score
Age		
2–20 years	–	–
20–40 years	1	1
> 40 years	2	2
Bleeding		
1–2 parts	1	1
3–4 parts	2	2
5–6 parts	3	3
AIDS	2	3
Edema	1	1
Icterus	1	1
Dyspnea	1	1
Bacterial infection	1	1
Leukocytes level < 1,500/mm^3^	–	2
Platelets level < 50,000/mm^3^	–	3
Renal insufficiency^a^	–	3
Maximum score	11	20

^a^Low glomerular filtration rate (below 60 ml/min/mm^2^) or serum creatinine above upper levels for age

### Statistical analysis

The data analysis was performed using STATA version 16 (Stata Corp., College Station, TX, USA) and GraphPad Prism version 9.0 (GraphPad Software Inc., San Diego, USA). The frequencies of categorical variables were compared using the chi-square test or Fisher's exact test, as appropriate. Continuous data were analyzed using the Mann–Whitney U test. Odds ratio (OR) and 95% confidence intervals (95% CI) were calculated to investigate associations of signs and symptoms with outcomes.

Multiple logistic regression analysis was performed to estimate adjusted odds ratios and 95% confidence intervals. The dependent variables were VL relapse (Model 1) and hospital death (Model 2). The independent variables included in logistic model 1 were age, sex, duration of antiretroviral use, splenomegaly, and adenomegaly. The independent variables included in the logistic model 1 were age, sex, duration of antiretroviral use, emaciation, edema, dehydration, general status, and paleness. The significance level adopted was 5%.

## Results

The Data on the general characterization of the sample are presented in Table 2. Most of the coinfected patients were male (89.3%). We observed that 41.4% had VL relapse and 11.2.% died during follow-up. The most frequent age group was 30 to 39 years (40.2%) and the majority self-reported their skin color as brown (68.1%). Regarding VL treatment, it was observed that liposomal amphotericin B was the medication most used (72.8%), the most frequent duration of medication use was up to 10 days (78.7%).

**Table 2:** Frequencies of visceral leishmaniasis relapse and death according to general variables and drug therapy

Variables	Total (n = 169)		Follow-up adverse outcomes					
			VL relapse (n = 70, 41.4%)		*P*	Death (n = 19, 11.2%)		*P*
			Yes	No		Yes	No	
	n	(%)	(%)	(%)		(%)	(%)	
Sex					.461			1.000
Male	151	(89.3)	(42.4)	(57.6)		(11.3)	(88.7)	
Female	18	(10.7)	(33.3)	(66.7)		(11.1)	(88.9)	
Age group					.120			.973
19 to 29 years	40	(23.7)	(30.0)	(70.0)		(10.0)	(90.0)	
30 to 39 years	68	(40.2)	(42.7)	(57.3)		(11.8)	(88.2)	
40 to 49 years	47	(27.8)	(53.2)	(46.8)		(10.6)	(89.4)	
50 years or more	14	(8.3)	(28.6)	(71.4)		(14.3)	(85.7)	
VL drug therapy					.156			.585
Liposomal amphotericin B	123	(72.8)	(46.3)	(53.7)		(13.0)	(87.0)	
Amphotericin B deoxycholate	27	(16.0)	(33.3)	(66.7)		(3.7)	(96.3)	
Meglumine antimoniate	10	(5.9)	(20.0)	(80.0)		(10.0)	(90.0)	
More than one drug	9	(5.3)	(22.2)	(77.8)		(11.1)	(88.9)	
Duration of medication use					.130			.343
Up to 10 days	133	(78.7)	(45.1)	(54.9)		(12.8)	(87.2)	
11 to 30 days	29	(17.2)	(31.0)	(69.0)		(3.4)	(96.6)	
More than 30 days	7	(4.1)	(14.3)	(85.7)		(14.3)	(85.7)	
Length of treatment					.355			.108
Less than 1 month	150	(88.8)	(43.3)	(56.7)		(12.0)	(88.0)	
1 to 6 months	16	(9.4)	(25.0)	(75.0)		(0)	(100)	
More than 6 months	3	(1.8)	(33.3)	(66.7)		(33.3)	(66.7)	
Antiretroviral therapy					.007			.323
3TC + TNF + LPV/RTV	56	(33.1)	(55.4)	(44.6)		(14.3)	(85.7)	
3TC + TNF + EFV	78	(46.2)	(34.6)	(65.4)		(10.3)	(89.7)	
3TC + TNF + ATV + RTV	13	(7.7)	(15.4)	(84.6)		(0)	(100)	
3TC + DDI + LPV/RTV	15	(8.9)	(60.0)	(40.0)		(6.7)	(93.3)	
Other	7	(4.1)	(14.3)	(85.7)		(28.6)	(71.4)	
Duration of antiretroviral use					< .001			.650
Up to 6 months	123	(72.8)	(52.0)	(48.0)		(10.6)	(89.4)	
More than 6 months	46	(27.2)	(13.0)	(87.0)		(13.0)	(87.0)	
Previous VL relapse					< .001			.056
Yes	64	(37.9)	(95.3)	(4.7)		(17.2)	(82.8)	
No	105	(62.1)	(8.6)	(91.4)		(7.6)	(92.4)	

*VL* visceral leishmaniasis, *3TC* lamivudine, *TNF* tenofovir, *LPV/RTV* lopinavir/ritonavir, *EFV* efavirenz, *ATV* atazanavir, *RTV* ritonavir, *DDI* didanosine

Regarding antiretroviral therapy, most of the patients was under treatment with lamivudine, tenofovir and efavirenz (46.2%) or lamivudine, tenofovir and lopinavir/ritonavir (33.1%) schemes. It was noticed that 72.8% of the sample were under antiretroviral therapy in the period up to 6 months. Association analysis showed that the patients on the lamivudine, didanosine and lopinavir/ritonavir regimen had higher relapse frequency (*P* = 0.007). The patients on antiretroviral therapy for up to 6 months had higher relapse frequency (*P* < 0.001). In addition, VL relapse was higher in patients who had previous relapse episodes (95.3% *versus* 8.6%, P < 0.001).

Table 3 presents the association analysis on VL signs and symptoms in relation to occurrences of relapse and death. These data were measured at the time of patient admission. Splenomegaly (OR = 2.20; 95% CI = 1.13–4.37) and adenomegaly (OR = 3.11; 95% CI = 1.02–10.52) were associated with a greater chance of VL relapse. Detection of weight loss (OR = 3.83; 95% CI = 1.15–17.11) and edema (OR = 4.05; 95% CI = 1.13–13.17) on physical examination were associated with the occurrence of death.

**Table 3:** Associations of signs and symptoms with visceral leishmaniasis relapse and death

Variables	Follow-up adverse outcomes			
	VL relapse		Death	
	OR (95% CI)	*p* value	OR (95% CI)	*p* value
Disease history data				
Fever	1.35 (.72–2.53)	.335	1.10 (.41–3.04)	.832
Hair loss	.80 (.28–2.16)	.667	–	.133
Diarrhea	1.06 (.57–1.98)	.828	.83 (.30–2.20)	.707
Vomiting	.97 (.46–2.03)	.954	.87 (.23–2.70)	1.000
Bleeding	1.27 (.55–2.91)	.555	.56 (.12–2.57)	.743
Cough	1.16 (.59–2.27)	.658	.83 (.25–2.39)	.740
Dyspnea	.87 (.44–1.71)	.702	1.80 (.65–4.84)	.229
Increased abdominal volume	1.00 (.51–1.94)	.987	1.31 (.45–3.56)	.584
Physical evaluation				
Emaciation	.93 (.49–1.75)	.832	3.83 (1.15–17.11)	.043
Febrile	1.03 (.49–2.16)	.922	1.69 (.55–4.77)	.313
Jaundice	.75 (.31–1.73)	.502	1.96 (.58–5.87)	.225
Alopecia	.44 (.09–1.65)	.362	–	.364
Spotting	1.75 (.49–6.49)	.528	1.83 (.25–8.52)	.356
Bleeding	1.26 (.41–3.76)	.665	2.14 (.44–8.05)	.381
Edema	.75 (.24–2.13)	.588	4.05 (1.13–13.17)	.012
Dyspnea	.61 (.18–1.84)	.385	.50 (.02–3.09)	1.000
Abdominal protrusion	1.16 (.57–2.34)	.669	.76 (.20–2.33)	.784
Hepatomegaly	.81 (.41–1.61)	.557	1.60 (.52–5.89)	.592
Splenomegaly	2.20 (1.13–4.37)	.018	1.66 (.58–5.39)	.346
Adenomegaly	3.11 (1.02–10.52)	.037	.54 (.02–3.36)	1.000

*VL* visceral leishmaniasis, *OR* odds ratio, *95% CI* 95% confidence interval

**Fig. 1:**
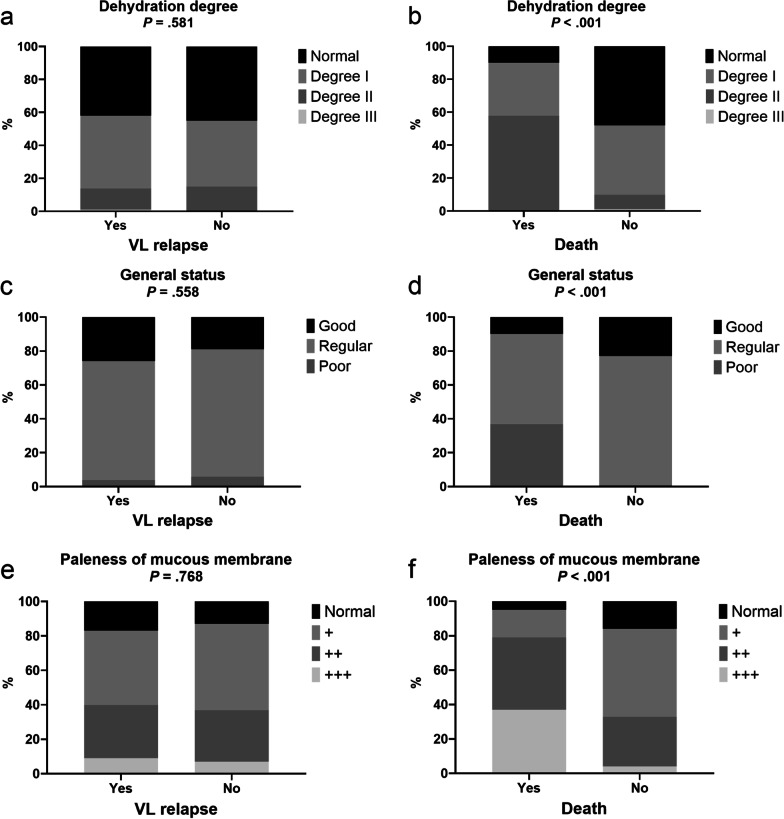
Frequencies of different degrees of dehydration (**a**, **b**), general status **c**, **d** and paleness of mucous membrane **e**, **f** according to visceral leishmaniasis relapse and death by the time of the follow-up. *VL* visceral leishmaniasis

Figure 1 shows the evaluation of dehydration, general condition and mucosal pallor according to the outcomes of relapse and death. No associations between these variables and VL relapse were observed. On the other hand, we noticed higher frequencies of dehydration, poor general health status and mucosal pallor among patients who had died by the time of the follow-up.

Table 4 presents the serum markers that showed associations with VL relapse. Patients with relapse had statistically higher levels of urea (*p* = 0.005) and creatinine (*p* < 0.001). Patients who died had statistically lower counts for red blood cells (*p* = 0.012), hemoglobin (*p* = 0.017) and platelets (*p* < 0.001).

**Table 4:** Median and interquartile range of serum biomarkers according to visceral leishmaniasis relapse and death by the time of the follow-up

Variables	VL relapse		*p* value	Death		*p* value
	Yes	No		Yes	No	
	Median [IQR]	Median [IQR]		Median [IQR]	Median [IQR]	
Red blood cells (million/mm^3^)	3.23 [2.76–3.67]	3.19 [2.58–3.65]	.884	2.77 [2.33–3.36]	3.27 [2.73–3.70]	.012
Hemoglobin (g/dl)	8.95 [7.70–10.80]	8.70 [7.40–10.20]	.472	7.90 [6.70–8.70]	8.90 [7.60–10.70]	.017
Hematocrit (%)	26.7 [23.2–32.8]	26.4 [22.6–31.6]	.686	24.3 [20.0–27.8]	26.8 [23.1–32.0]	.054
Leukocytes (1000/mm^3^)	2.08 [1.44–3.88]	2.30 [1.60–3.73]	.421	2.04 [1.37–4.02]	2.26 [1.55–3.77]	.790
Platelets (1000/mm^3^)	120.5 [82.0–150.0]	121.0 [87.0–186.0]	.510	52.5 [33.0–81.0]	155.0 [122.0–215.0]	< .001
AST (U/l)	41.0 [28.0–60.0]	41.0 [27.0–59.0]	.640	48.0 [37.0–76.0]	40.0 [27.0–59.0]	.211
ALT (U/l)	28.5 [18.0–63.0]	31.0 [20.0–55.0]	.606	24.0 [17.0–43.0]	29.0 [20.0–46.0]	.331
Urea (mg/dl)	39.5 [27.5–51.5]	32.0 [23.0–40.0]	.005	44.5 [28.0–56.0]	34.0 [25.0–45.0]	.122
Creatinine (mg/dl)	1.02 [.84–1.23]	.76 [.56–0.93]	< .001	1.03 [.69–1.36]	.87 [.67–1.09]	.182

*VL* visceral leishmaniasis, *IQR* interquartile range [1st quartile–3rd quartile], *AST* aspartate aminotransferase, *ALT* alanine aminotransferase

**Fig. 2:**
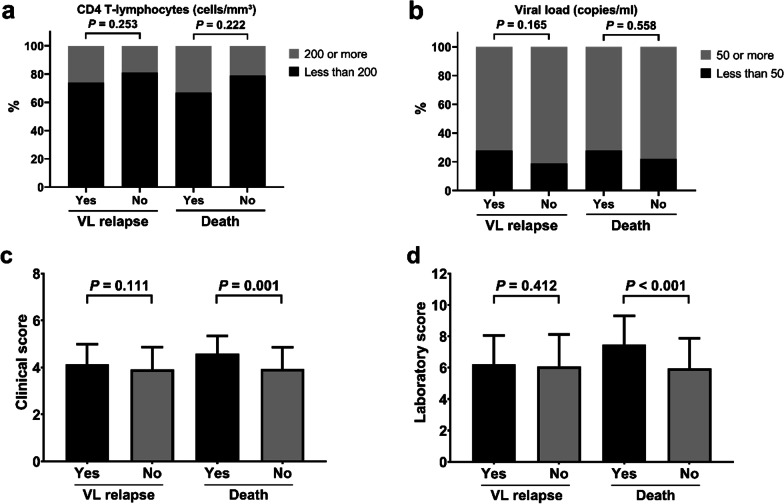
Frequencies of categories of CD4 T lymphocytes (**a**) and viral load (**b**), and mean with standard deviation of clinical score (**c**) and laboratory score (**d**), according to VL relapses and deaths by the time of the follow-up. *VL* visceral leishmaniasis

Figure 2 shows that CD4 T-lymphocyte level, viral load, clinical score and laboratory score according to the outcome did not present any association with relapse and death. Clinical and laboratory scores were not significantly associated with relapse. On the other hand, clinical scores (*p* = 0.001) and laboratory scores (*p* < 0.001) were statistically significantly higher among patients who died.

The multiple logistic regression analysis is shown in Table 5. Antiretroviral therapy more than 6 months was associated with a decrease in VL relapse (adjusted OR = 0.10, 95% CI = 0.03-0.31, p < 0.001). Adenomegaly was associated with an increase in VL relapse (adjusted OR = 4.65, 95% CI = 1.11–19.45, p = 0.035). In model 2, edema (adjusted OR = 12.36, 95% CI = 2.59–58.78, p = 0.001), dehydration (6.36, 95% CI = 1.51–26.69, p = 0.011), poor general health status (adjusted OR = 33.08, 95% CI = 4.46–245, p < 0.001), and paleness (adjusted OR = 4.78, 95% CI = 1.12–20.39, p = 0.034) were associated with an increase in hospital death.

**Table 5:** Multiple logistic regression model of clinical factors on the VL relapse and hospital death

Variables	Adjusted OR	(95% CI)	*p* value
Outcome: VL relapse			
Antiretroviral use (more than 6 months)	.10	(.03–0.31)	< .001
Splenomegaly	1.81	(.84–3.90)	.129
Adenomegaly	4.65	(1.11–19.45)	.035
Outcome: hospital death			
Emaciation	1.15	(.23–5.63)	.862
Edema	12.36	(2.59–58.78)	.001
Dehydration (degree II/III)	6.36	(1.51–26.69)	.011
General health status (poor)	33.08	(4.46–245)	.000
Paleness (+ + / + + +)	4.78	(1.12–20.39)	.034

*VL* visceral leishmaniasis, *OR* odds ratio, *95% CI* 95% confidence interval. Model 1 was adjusted for age, sex, duration of antiretroviral use, splenomegaly, and adenomegaly. Model 2 was adjusted for age, sex, duration of antiretroviral use, emaciation, edema, dehydration, general status, and paleness

## Discussion

The main findings from the present study indicated that the percentage of VL relapse was 41.4% among VL/HIV coinfected patients in this city in northeastern Brazil. In a study conducted in the state of Ceará, Brazil, 29% of coinfected patients had an unfavorable outcome, which was recurrence in 21.4% [13]. A study found that the propensity to adverse events and death was three times higher among coinfected patients than among HIV-negative patients [14]. According to Lindoso et al. [15], patients coinfected with VL/HIV were at increased risk of relapse and lethality, which was clearly determined by the preponderance of the immune response, and mainly by the CD4 + T lymphocyte count. It is important to notice that Maranhão, the state where the present study was conducted, is the second state in Brazil with the highest percentage of VL patients [2, 3].

The data from the present study showed that there were higher frequencies of coinfected men and individuals in the age group of 30 to 39 years. These results were in agreement with other data in the literature, which may reflect the fact that HIV and leishmaniasis are proportionally more prevalent among the male population than in the general population [16].

A study has shown that male sex was a potential risk factor for seroprevalence, seroconversion and incidence [17]. This strongly suggested that biological factors such as the role of hormones in modulating the immune system may be related to sex in the pathogenesis of leishmaniasis. Evidence has shown that testosterone was associated with increase *L. donovani* uptake by macrophages, thereby increasing the infection rates and levels of these cells in vitro, which suggested that this hormone had a direct influence on increasing the level of infected cells [17]. Other studies indicate that there are no definitive conclusions about the reasons for the disparity between the sexes, and that this difference between men and women may be associated with reasons other than hormonal factors, such as the exclusion of some groups that may affect the results, for example, pregnant women [18].

Regarding VL treatment, it was observed in the present study that liposomal amphotericin B was the most prescribed drug therapy (72.8%), which is in line with the guidelines of the World Health Organization [19, 20] and the Brazilian Ministry of Health [9], which recommends amphotericin B as the first-choice therapy for VL/HIV coinfected patients. This drug can be used in liposomal form or as amphotericin B deoxycholate. Liposomal amphotericin B is the main drug used worldwide, because of its better outcomes. It is the most potent leishmanicidal agent that is commercially available, and it also has the advantage of low toxicity, compared with conventional amphotericin B [15].

In this study, liposomal amphotericin B was the drug that had the highest number of relapses and deaths. Studies show that liposomal amphotericin B has some side effects, including decreased potassium and transient changes in creatinine levels [21]. In Brazil, it is the most widely used drug because it is the drug of the first choice indicated and made available by the country's health system [9]. Thus, more studies would be needed to know if there is a correlation between the number of relapses and deaths and the drug used [21].

Most of the sample was receiving highly active antiretroviral therapy (HAART) consisting of a regimen of either lamivudine + tenofovir + efavirenz (3TC + TDF + EFZ) (46.2%) or lamivudine + tenofovir + lopinavir/ritonavir (3TC + TDF + LPV/RTV) (33.1%), and it was noticed that 72.8% had only started the therapy within the last six months. In this study, patients on antiretroviral therapy for up to six months had statistically higher relapse frequency. This may be explained by the lack of complete recovery of CD4 + counts in these individuals. Even after antiretroviral therapy has been started, a certain degree of stabilization is still required for the immune system to be able to respond adequately again [22]. Davi-Mendez et al. [22] stated that a CD4/CD8 ratio < 1 was associated with biomarkers of activation and inflammation, and was predictive of morbidity and even mortality due to non-AIDS-related causes. In their study, the average time taken for normalization of the CD4/CD8 ratio was six months. One year of starting the therapeutic scheme, 62% of patients had recovered, while 38% still had abnormal CD4 + levels. Thus, VL/HIV coinfected patients with only up to six months of antiretroviral therapy may be more susceptible to relapse episodes.

Parasitic infection together with viral infection induces chronic immune activation, which promotes increased HIV load, accelerated progression to AIDS and presence of immunological disturbances that propitiate uncontrolled parasite multiplication. Patients with decreased CD4 + cell counts (< 200 cells/mm^3^) have higher frequency of VL clinical manifestations [14, 15, 23].

It was observed that 11.2% of the patients died during the follow-up of this study. This proportion was considered low in comparison with the mortality rates among coinfected patients described in other studies. It more closely resembled the mortality rates of HIV-positive people without coinfection [24]. However, the present study provided the first description of the hospital death rate among VL/HIV coinfected patients, which may serve as an explanation for the differences in the numbers found.

Santos et al. [14] found a mortality rate of 24.3%, a rate similar to that found by Sousa-Gomes et al. [6] (23.2%). Both of these studies were conducted using database records available from the Brazilian Ministry of Health. Guedes et al. [16], in a study conducted in Pernambuco (northeastern Brazil), found a mortality rate of 14.3%, and Távora et al. [13] obtained a rate of 7.14%.

Some statistically significant associations were observed with regard to the physical examination variables. Splenomegaly and adenomegaly were associated with a greater chance of VL relapse. Hurissa et al. [25] studied 92 coinfected patients; in their results, all patients presented splenomegaly and generalized weakness. In a study by Mohammed et al. [26] among groups of VL/HIV coinfected patients who presented repeated relapses, the frequency of spleen enlargement was 98%, which may indicate a form of primary host immune deficit leading to multiple relapses.

Detection of weight loss and edema in the physical examination was associated with occurrence of death. Although some studies have shown an atypical clinical manifestation in coinfected patients, other studies corroborate the present study through showing classic symptoms such as fever, weight loss and splenomegaly [23, 27]. On the other hand, we noted that the frequencies of greater-impairment categories of these variables were higher among patients who had died by the time of the follow-up of the study.

In addition, present data showed that previous episodes of VL relapse were associated with new relapse. This finding can be supported by some studies that have suggested that Th2-mediated immune disorders could affect later infection control [29, 30].

In the present study, patients with relapsing VL had statistically significantly higher levels of urea and creatinine. Other authors have also found abnormal creatinine levels in their studies with VL/HIV coinfected patients [13, 28]. The probable explanation for this is that the associations of drugs that are used to treat HIV and related infections are responsible for a substantial proportion of the renal abnormalities developed in this group of patients. Both HAART and conventional amphotericin B have extensive renal toxicity, which leads to several serum changes [31].

The present findings showed that patients who died had statistically lower counts for red blood cells, hemoglobin and platelets. Anemia has already been reported among HIV-negative VL patients: its cause is probably multifactorial and may include immune-mediated mechanisms, changes to red blood cell membrane permeability, hypersplenism and hemolysis. Henn et al. [32] found significantly lower hemoglobin and lymphocyte levels in their sample. The ability to modify the host immune response can be considered to be one of the main factors leading to thrombocytopenia [13, 27]. In addition, studies have shown that even after reasonable levels of CD4 counts and viral suppression of HIV have been reached, coinfected patients do not keep the parasite under control, even months after starting antiretroviral therapy [26].

## Conclusion

The study showed a high frequency of relapses among VL/HIV coinfected patients, being associated with splenomegaly, adenomegaly and elevated urea and creatinine levels. The clinical variables of weight loss and edema and the laboratory variables of anemia and thrombocytopenia were associated with the outcome death. These data demonstrate the need to include other variables in the predictive models of prognosis when we talk about coinfected patients, with the need to consider the physical examination in these models focusing on the search for signs and symptoms associated with severe outcomes such as splenomegaly, adenomegaly, weight loss, and edema. These data are even more important since coinfection is more prevalent in low- and middle-income countries where resources to perform complex exams are scarcer and where physical examination becomes a powerful tool to reduce recurrence and mortality in this population.

## Data Availability

The datasets used and/or analysed during the current study are available from the corresponding author on reasonable request.

## References

[1] Azevedo TS, Lorenz C, Chiaravalloti-Neto F (2019). Risk mapping of visceral leishmaniasis in Brazil. Rev Soc Bras Med Trop.

[2] Pan American Health Organization. Leishmaniasis: Epidemiological Report in the Americas. Number 9, December 2020. Washington, D.C.: PAHO, 2020. Available at: https://iris.paho.org/handle/10665.2/53090. Accessed 25 May 2021.

[3] Ministério da Saúde, Departamento de Informática do Sistema Único de Saúde. Informações de Saúde (TABNET): Epidemiológicas e Morbidade. Available at: http://www2.datasus.gov.br/DATASUS/index.php?area=0203. Accessed 25 May 2021.

[4] Lima URS, Vanolli L, Moraes EC, Ithamar JS, Azevedo CMPES (2019). Visceral leishmaniasis in Northeast Brazil: What is the impact of HIV on this protozoan infection?. PLoS ONE.

[5] Ministério da Saúde, Secretaria de Vigilância em Saúde. Departamento de Vigilância das Doenças Transmissíveis. Manual de recomendações para diagnóstico, tratamento e acompanhamento de pacientes com a coinfecção leishmania–HIV. Brasília, 2015. http://bvsms.saude.gov.br/bvs/publicacoes/manual_recomendacoes_diagnostico_leishmania_hiv.pdf. Accessed 25 May 2021.

[6] Sousa-Gomes ML, Romero GAS, Werneck GL (2017). Visceral leishmaniasis and HIV/AIDS in Brazil: are we aware enough?. PLoS Negl Trop Dis.

[7] Viana GMC, Silva MACN, Garcia JVS (2017). Epidemiological profile of patients co–infected with visceral leishmaniasis and HIV/AIDS in Northeast. Brazil Rev Soc Bras Med Trop.

[8] Ministério da Saúde, Secretaria de Vigilância em Saúde. Departamento de Vigilância, Prevenção e Controle das Infecções Sexualmente Transmissíveis, do HIV/Aids e das Hepatites Virais. Protocolo Clínico e Diretrizes Terapêuticas para Manejo da Infecção pelo HIV em Adultos, 2018. Available at: http://www.aids.gov.br/pt-br/pub/2013/protocolo-clinico-e-diretrizes-terapeuticas-paramanejo-da-infeccao-pelo-hiv-em-adultos. Accessed 25 May 2021.

[9] Ministério da Saúde, Secretaria de Vigilância em Saúde. Departamento de Vigilância Epidemiológica. Leishmaniose visceral grave: normas e condutas. Brasília: Ministério da Saúde, Secretaria de Vigilância em Saúde; 2006.

[10] Costa DL, Rocha RL, Chaves EB, Batista VG, Costa HL, Costa CH (2016). Predicting death from kala-azar: construction, development, and validation of a score set and accompanying software. Rev Soc Bras Med Trop.

[11] Porto CC (2008). Exame clínico.

[12] Ministério da Saúde, Secretaria de vigilância em saúde. Departamento de vigilância epidemiológica. Manual de vigilância e controle da leishmaniose visceral. 1 ed. 5º reimpressão. Brasília, 2014. http://bvsms.saude.gov.br/bvs/publicacoes/manual_vigilancia_controle_leishmaniose_visceral_1edicao.pdf. Accessed 25 May 2021.

[13] Távora LG, Nogueira MB, Gomes ST (2015). Visceral Leishmaniasis/HIV co–infection in northeast Brazil: evaluation of outcome. Braz J Infect Dis.

[14] Santos GO, Jesus NPS, Cerqueira-Braz JV, Santos VS, Lemos LMD (2019). Prevalence of HIV and associated factors among visceral leishmaniasis cases in an endemic area of Northeast Brazil. Rev Soc Bras Med Trop.

[15] Lindoso JAL, Moreira CHV, Cunha MA, Queiroz IT (2018). Visceral leishmaniasis and HIV coinfection: current perspectives. HIV AIDS.

[16] Guedes DL, Medeiros Z, Silva ED (2018). Visceral leishmaniasis in hospitalized HIV-infected patients in Pernambuco. Brazil Am J Trop Med Hyg.

[17] Cloots K, Burza S, Malaviya P (2020). Male predominance in reported Visceral Leishmaniasis cases: Nature or nurture? A comparison of population–based with health facility–reported data. PLoS Negl Trop Dis.

[18] Dahal P, Singh-Phulgenda S, Olliaro PL, Guerin PJ (2021). Gender disparity in cases enrolled in clinical trials of visceral leishmaniasis: a systematic review and meta-analysis. PLoS Negl Trop Dis..

[19] Zhang H, Zhao J, Wang P, Qiao Z (2001). Effect of testosterone on Leishmania donovani infection of macrophages. Parasitol Res.

[20] World Health Organization. Control of the leishmaniases: report of a meeting of the WHO Expert Commitee on the Control of Leishmaniases, Geneva, 2010. https://apps.who.int/iris/handle/10665/44412. Accessed 25 May 2021.

[21] Yang YL, Xiang ZJ, Yang JH, Wang WJ, Xu ZC, Xiang RL. Adverse Effects Associated With Currently Commonly Used Antifungal Agents: A Network Meta-Analysis and Systematic Review. Front Pharmacol. 2021 https://www.ncbi.nlm.nih.gov/pmc/articles/PMC8585744/. Accessed 28 May 2022. 10.3389/fphar.2021.697330 PMC8585744 34776941

[22] Davy-Mendez T, Napravnik S, Zakharova O (2018). Acute HIV infection and CD4/CD8 ratio normalization after antiretroviral therapy initiation. J Acquir Immune Defic Syndr.

[23] Alvar J, Cañavate C, Gutiérrez-Solar B (1997). Leishmania and human immunodeficiency virus coinfection: the first 10 years. Clin Microbiol Rev.

[24] Croxford S, Kitching A, Desai S (2017). Mortality and causes of death in people diagnosed with HIV in the era of highly active antiretroviral therapy compared with the general population: an analysis of a national observational cohort. Lancet Publ Heal.

[25] Hurissa Z, Gebre-Silassie S, Hailu W (2010). Clinical characteristics and treatment outcome of patients with visceral leishmaniasis and HIV co–infection in northwest Ethiopia. Trop Med Int Health.

[26] Mohammed R, Fikre H, Schuster A, Mekonnen T, Griensven JV, Diro E (2020). Multiple relapses of visceral leishmaniasis in HIV Co–Infected Patientes: a case series from Ethiopia. Curr Ther Res.

[27] Fontoura I, Barbosa D, Andrade PAD (2018). Epidemiological, clinical and laboratory aspects of human visceral leishmaniasis (HVL) associated with human immunodeficiency virus (HIV) coinfection: a systematic review. Parasitol.

[28] Mahajan R, Das P, Isaakidis P (2015). Combination treatment for visceral leishmaniasis patients coinfected with human immunodeficiency virus in India. Clin Infect Dis.

[29] Cota GF, De Sousa MR, Rabello A (2011). Predictors of visceral leishmaniasis relapse in HIV-infected patients: a systematic review. PLoS Negl Trop Dis.

[30] Diro E, Ritmeijer K, Boelaert M (2015). Use of pentamidine as secondary prophylaxis to prevent visceral leishmaniasis relapse in HIV infected patients, the first twelve months of a prospective cohort study. PLoS Negl Trop Dises.

[31] Venter WDF, Fabian J, Feldman C (2018). An overview of tenofovir and renal disease for the HIV-treating clinician. South Afr J HIV Med.

[32] Henn GAL (2018). Is Visceral Leishmaniasis the same in HIV–coinfected adults?. Braz J Infect Dis.

